# Combination treatment with fingolimod and a pathogenic antigen prevents relapse of glucose‐6‐phosphate isomerase peptide‐induced arthritis

**DOI:** 10.1002/iid3.111

**Published:** 2016-06-08

**Authors:** Yuya Yoshida, Norihisa Mikami, Yuki Matsushima, Mai Miyawaki, Hiroki Endo, Rie Banno, Takumi Tsuji, Tetsuro Fujita, Takeyuki Kohno

**Affiliations:** ^1^ Faculty of Pharmaceutical Sciences, Department of Pathological BiochemistrySetsunan UniversityOsakaJapan; ^2^ Department of Experimental ImmunologyImmunology Frontier Research CenterOsaka UniversityOsakaJapan; ^3^ Research Institute for Production and DevelopmentKyotoJapan

**Keywords:** FTY720, immune tolerance, rheumatoid arthritis

## Abstract

**Introduction:**

Combination treatment with fingolimod (FTY720) plus pathogenic antigen is thought to prevent glucose‐6‐phosphate isomerase (GPI)_325‐339_‐induced arthritis progression by effective induction of immune tolerance. Here, we examined the efficacy of this combination treatment on remission maintenance.

**Methods:**

GPI_325‐339_‐induced arthritis mice were treated for 5 days with FTY720 (1.0 mg/kg, *p.o*.) alone, GPI_325–339_ (10 μg/mouse, *i.v*.) alone, or with the FTY720 plus GPI_325‐339_ combination. In some experiments, mice were resensitized with GPI_325‐339_.

**Results:**

Following resensitization with GPI_325‐339_, combination‐treated mice exhibited neither severe relapse nor elevated lymphocyte infiltration in joints. Neither anti‐human nor mouse GPI_325‐339_ antibody levels were correlated with clinical symptoms. This suggests that combination treatment prevents relapse following resensitization *via* regulation of pathogenic antigen‐specific T cells. The proportion of regulatory T (Treg) cells in inguinal lymph nodes was increased post treatment in the FTY720 alone and FTY720 plus GPI_325‐339_ groups. In contrast, the proportion of glucocorticoid‐induced tumor necrosis factor receptor‐family‐related gene/protein (GITR)^+^ non‐Treg cells was increased only in combination‐treated mice. Furthermore, GITR^+^ non‐Treg cells, which were induced by the combination treatment *in vivo*, possess suppressive activity and high ability to produce interleukin (IL)‐10.

**Conclusion:**

GITR^+^ non‐Treg cells might play a key role in relapse prevention following resensitization. Thus, this combination treatment might establish immune tolerance by induction of GITR^+^ non‐Treg cells.

**Abbreviations**
BSA: bovine serum albumin
CTLA‐4: cytotoxic T‐lymphocyte antigen 4
Foxp3: Fork head box P3
FTY720‐P: FTY720 monophosphate
GITR: glucocorticoid‐induced tumor necrosis factor receptor‐family‐related gene/protein
GPI: glucose‐6‐phosphate isomerase
IgG: immunoglobulin G
IL: interleukin
iTreg: induced Treg
LN: lymph node
mAb: monoclonal antibody
nTreg: natural Treg
PE: phycoerythrin
RA: rheumatoid arthritis
Treg: regulatory T
Tr1: Type 1 regulatory T

## Introduction

Rheumatoid arthritis (RA) is a systemic autoimmune disease characterized by joint inflammation and cartilage destruction. The symptoms of RA are generally typified by repeating cycles of exacerbation and remission. The induction of clinical remission has been the primary focus of RA treatment in recent years. Clinical remission is facilitated by biological agents such as infliximab, etanercept, adalimumab, tocilizumab, early diagnosis, and management. However, further studies are required to identify novel approaches for drug‐free remission. Initial studies on the induction of drug‐free remission have been conducted with several agents including infliximab 1 and adalimumab 2. For example, a previous report has shown that 56/102 (55%) RA patients, who achieved low disease activity using infliximab, were able to discontinue infliximab for over a year without progression of radiological articular destruction 1. Thus, the development of new treatment strategies is required to improve the rate of complete remission.

Fingolimod or FTY720, is a novel immunosuppressant discovered by Fujita et al.; it is a synthetic structural analogue of myriocin (ISP‐I), a compound derived from *Isaria sinclairii* 3, 4. In September 2010, FTY720 was approved by the United States Food and Drug Administration for the treatment of relapsing remitting multiple sclerosis. FTY720 has been reported to be effective in several immunological disease models, including multiple sclerosis 5, myasthenia gravis 6, atopic dermatitis 7, 8, and type 1 diabetes mellitus 9, 10. The unique mode of action of FTY720 differs from that of established immunosuppressants, such as tacrolimus hydrate and cyclosporine; FTY720 is converted *in vivo* by sphingosine kinase 2 to FTY720 monophosphate (FTY720‐P), which is the active form of the drug. FTY720‐P acts as a high‐affinity agonist of four sphingosine 1‐phosphate receptors (S1P_1_, S1P_3_, S1P_4_, and S1P_5_) 11; FTY720‐P induces long‐term down‐regulation of S1P_1_ in lymphocytes and suppresses immune responses by sequestering circulating mature lymphocytes from blood and peripheral tissues to secondary lymphoid tissues and the thymus 12, 13. Combination treatment with FTY720 plus pathogenic antigens, such as glucose‐6‐phosphate isomerase peptide (GPI_325‐339_), efficiently suppresses symptom progression of GPI_325‐339_‐induced arthritis, an animal model of RA, by inducing clonal deletion and anergy of pathogenic T cells as well as immune suppression *via* regulatory T (Treg) cells 14.

In the present study, we examined whether combination treatment with FTY720 plus pathogenic antigens would enable remission maintenance using the GPI_325‐339_‐induced arthritis mouse model.

## Results

### Combination treatment with FTY720 plus GPI_325‐339_ suppresses relapse following resensitization

**Figure 1:**
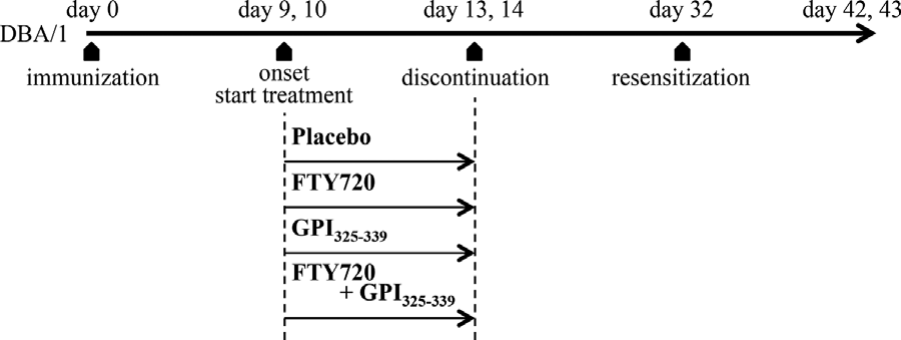
Study timeline DBA/1 mice were immunized with GPI_325‐339_ on day 0. GPI_325‐339_‐induced arthritis mice were treated from the day of arthritis onset (days 9–10) to days 13–14. In some experiments, mice were resensitized with GPI_325‐339_ on day 32. Clinical symptom evaluation and antibody titer measurements were performed on days 0–42 or 43 and 42–43, respectively.

**Figure 2:**
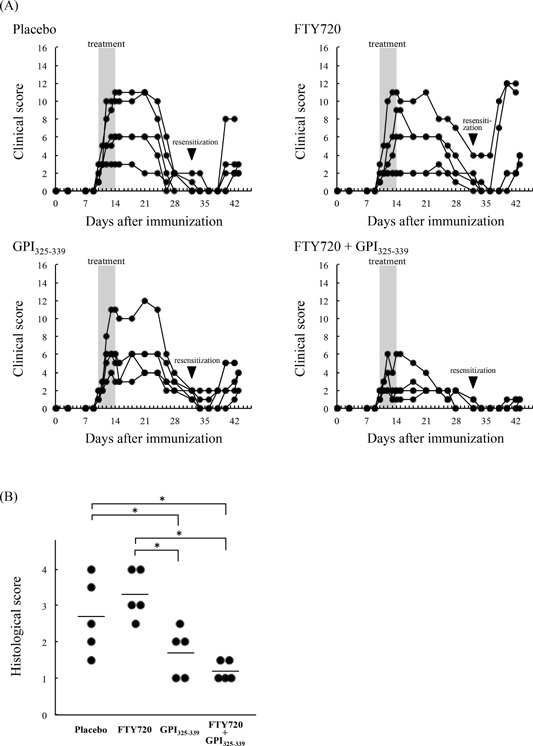
Relapse following resensitization was suppressed by combination treatment with FTY720 plus GPI_325‐339_. GPI_325‐339_‐induced arthritis mice were treated with FTY720 alone, GPI_325‐339_ alone, or FTY720 plus GPI_325‐339_ combination. At day 32 after the first immunization, mice were resensitized with hGPI_325‐339_. (A) Clinical symptoms of arthritis were evaluated from the day of first immunization to days 42–43 (*n* = 5 for each group). (B) At days 42–43 after the first immunization, joint tissues were excised and stained with hematoxylin‐eosin. Synovial hyperplasia and lymphocyte infiltration of joints were histologically graded. The mean for all mice (*n* = 5) in each group is indicated by the horizontal line. The significance of differences was examined by Duncan's test (*denotes *P *< 0.05).

In order to effect complete remission of an autoimmune disease, it is necessary to suppress the immune response as well as maintain the immunosuppressed state. To examine the effect of combination treatment with FTY720 plus GPI_325‐339_ on remission maintenance, mice treated with FTY720 alone, GPI_325‐339_ alone, or the FTY720 plus GPI_325‐339_ combination, were resensitized with GPI_325‐339_ at day 32 after the first immunization in order to induce a relapse (Fig. 1). Changes in clinical symptoms were determined based on appearance. Severe or moderate relapse occurred following resensitization in all animals of the placebo, FTY720 alone, and GPI_325‐339_ alone groups (Fig. 2A); relapse was still apparent at day 42–43 after the first immunization, although more prolonged observation is required. In contrast, combination treatment with FTY720 plus GPI_325‐339_ efficiently suppressed relapse following resensitization in all animals (*n* = 5); only low intensity symptoms were observed (Fig. 2A). In addition, synovial hyperplasia and lymphocyte infiltration were mild or absent in the combination treatment group (Fig. 2B). Although no significant difference was apparent between the histological scores of the FTY720 plus GPI_325‐339_ and GPI_325‐339_ alone groups, 3/5 mice in the GPI_325‐339_ alone group (with a histological score of 2.0–2.5; Fig. 2B), exhibited a moderate relapse (Fig. 2A). The histological scores correlated with the clinical scores at days 42–43 after the first immunization (*P* < 0.05, Pearson's correlation coefficient test).

### Combination treatment affects pathogenic T cells but not anti‐GPI_325‐339_ antibodies

**Figure 3:**
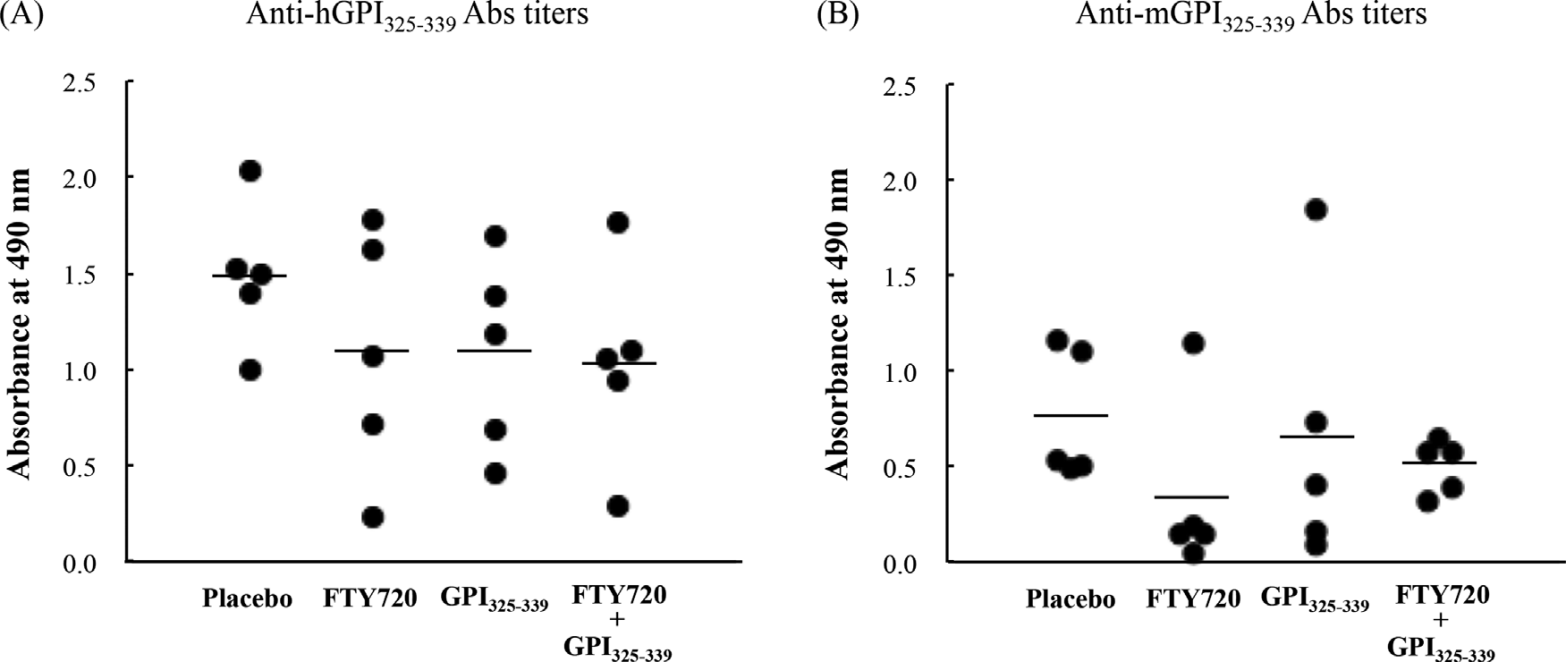
Anti‐GPI_325‐339_ antibodies might not constitute an important trigger of arthritis relapse. GPI_325‐339_‐induced arthritis mice were treated with FTY720 alone, GPI_325‐339_ alone, or the FTY720 plus GPI_325‐339_ combination. At day 32 after the first immunization, mice were resensitized with hGPI_325‐339_. At days 42–43 after the first immunization, peripheral blood samples were collected and anti‐(A) human or (B) mouse GPI_325‐339_ total IgG antibody titers were measured by enzyme‐linked immunosorbent assay. The mean for all mice (*n* = 5) in each group is indicated by the horizontal line. The significance of differences was examined by Duncan's test. No significant differences were found between each of the groups for either anti‐human or mouse GPI_325‐339_ total IgG antibody titers.

Schubert et al. 15 previously demonstrated that depletion of CD4^+^ cells by treatment with anti‐CD4 monoclonal antibody (mAb) prevented GPI‐induced arthritis. In addition, it has been reported that adoptive transfer of purified immunoglobulin G (IgG) from GPI‐induced arthritis mice failed to induce arthritis symptoms and that Fcγ receptor‐deficient mice were resistant to GPI‐induced arthritis 15. Iwanami et al. 16 previously suggested that GPI antibodies might play a role in the development of peptide‐induced arthritis, since cartilage surface‐deposited IgG was detected in GPI_325‐339_‐immunized mice. These findings indicate that the development of GPI_325‐339_‐induced arthritis may require both pathogenic T cells and anti‐GPI_325‐339_ antibodies. Therefore, we sought to confirm the effect of combination treatment on the production of the anti‐GPI_325‐339_ IgG antibody. To confirm the correlation between anti‐human or mouse GPI_325‐339_ IgG antibodies and relapse following resensitization, the level of anti‐human or mouse GPI_325‐339_ IgG antibodies was measured by enzyme‐linked immunosorbent assay. Both the anti‐human and mouse GPI_325‐339_ IgG antibodies exhibited considerable individual variability in all groups (Fig. 3). In addition, neither the anti‐human nor mouse GPI_325‐339_ IgG antibodies were correlated with the clinical and histological scores at days 42–43 after the first immunization (*P* *> *0.05, Pearson's correlation coefficient test). Thus, anti‐human and mouse GPI_325‐339_ IgG antibodies might not be involved directly in relapse post resensitization. Further studies will be required to establish the cause for the deposition of IgG on the cartilage surface.

### Combination treatment induces GITR and CD39‐expressing Treg cells in inguinal lymph nodes

**Figure 4:**
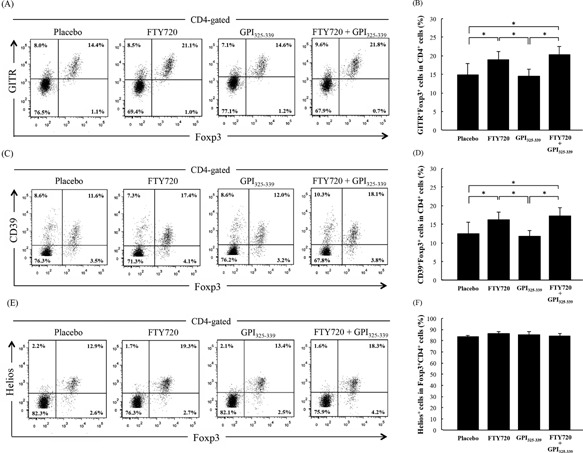
FTY720‐induced Treg cells in inguinal lymph nodes are natural Treg cells. Cells were obtained from inguinal lymph nodes of treated mice at day 14 (completion of treatment). The percentage of (A, B) GITR^+^Foxp3^+^ cells or (C, D) CD39^+^Foxp3^+^ cells in CD4^+^ T cells and (E, F) Helios^+^ cells in Foxp3^+^CD4^+^ T cells was determined by flow cytometric analysis. The results are shown as mean + SD (four animals from one experiment). The significance of differences was examined by Duncan's test (*denotes *P *< 0.05)

CD25^+^CD4^+^ Treg cells prevent autoimmune responses by balancing the immune system. Glucocorticoid‐induced tumor necrosis factor receptor‐family‐related gene/protein (GITR), also known as tumor necrosis factor receptor superfamily member 18, is expressed on CD25^+^CD4^+^ Treg cells and plays a key role in the maintenance of immunological self‐tolerance 17. In addition, it is known that CD39 and CD73‐expressing CD25^+^CD4^+^ Treg cells induce immune suppression by producing extracellular adenosine, which can engage the adenosine receptors on effecter T cells 18, 19. Therefore, in order to examine whether GITR‐expressing Fork head box P3 (Foxp3)^+^CD4^+^ cells and CD39‐expressing Foxp3^+^CD4^+^ cells are induced by combination treatment with FTY720 plus GPI_325‐339_, cells were collected from inguinal lymph nodes (LNs) upon treatment completion and analyzed by flow cytometry. A previous study reported that cytotoxic T‐lymphocyte antigen 4 (CTLA‐4)‐expressing Treg cells were increased following administration of FTY720 14. Our results show that the proportion of both GITR^+^Foxp3^+^ and CD39^+^Foxp3^+^ cells in CD4^+^ cells was increased in the FTY720 alone and the FTY720 plus GPI_325‐339_ groups (Fig. 4A–D).

Treg cells can be broadly divided into two types, natural Treg (nTreg) cells and induced Treg (iTreg) cells; nTreg cells are thought to be functionally stable in terms of epigenetic patterns 20. Therefore, in order to examine whether the Treg cells increased by FTY720 treatment are nTreg or iTreg cells, the proportion of Helios‐expressing Treg cells in inguinal LNs was determined by flow cytometry. Helios, also known as Ikaros family zinc finger 2, is a known specific marker for thymic‐derived Treg (nTreg) cells 21. No significant difference was observed between the percentage of Helios^+^ cells in Treg cells in the placebo group and the FTY720‐treated groups (FTY720 alone and FTY720 plus GPI_325‐339_; Fig. 4E, F). Thus, the ratio of Helios negative Treg cells, that is, iTreg cells, remained unchanged. These data suggest that the FTY720‐increased Treg cells are nTreg because nTreg cells constituted the majority of Treg cells (approximately 85%) in all groups (Fig. 4E, F), and that FTY720 could create a functional Treg‐rich environment in secondary lymphoid tissues. However, while relapse was observed in FTY720 alone treated mice following resensitization, no severe relapse was observed in the combination treatment group. These results indicate that additional factors contribute to relapse suppression in mice treated with a combination of FTY720 plus GPI_325‐339_.

### Combination treatment increases GITR‐expressing non‐Treg cells in inguinal lymph nodes

**Figure 5:**
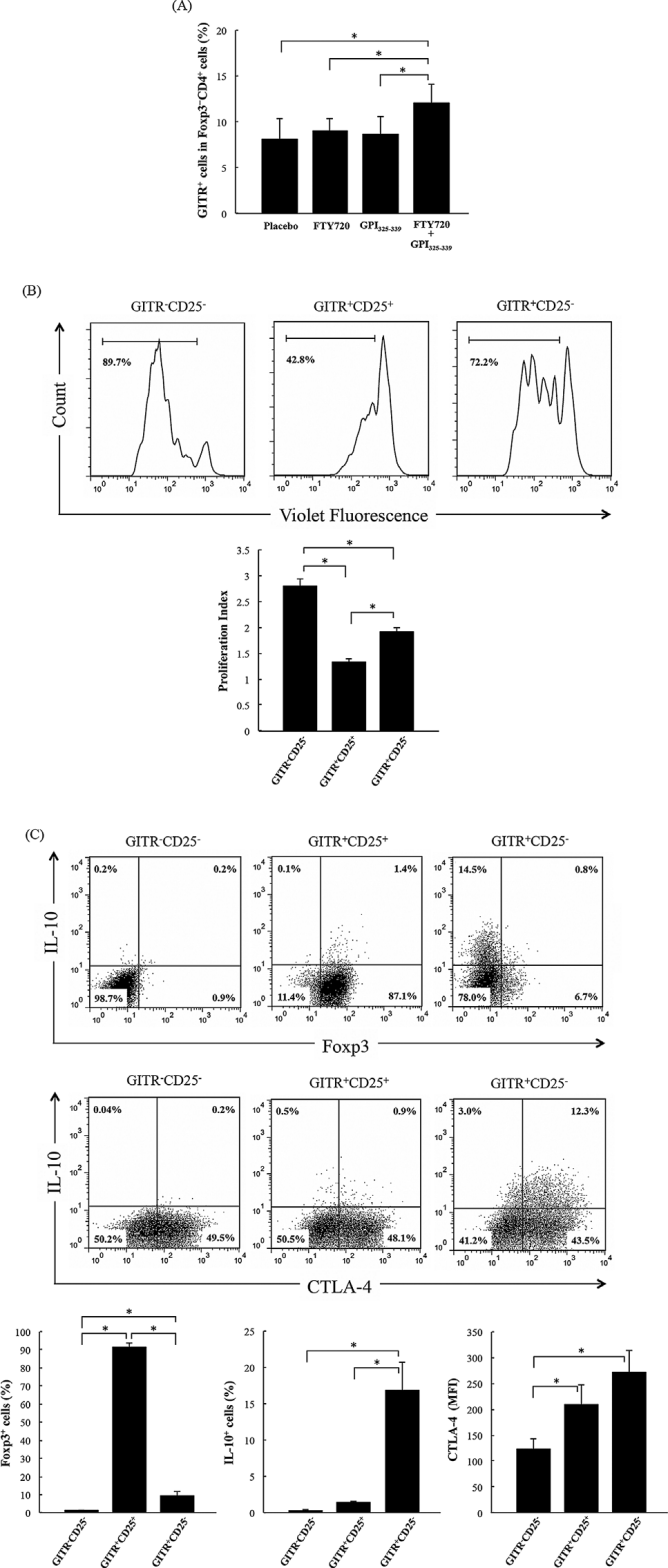
Combination treatment with FTY720 plus GPI_325‐339_ induces GITR^+^ non‐Treg cell populations in inguinal lymph nodes. Cells were obtained from inguinal lymph nodes of treated mice at day 14 (completion of treatment). (A) The percentage of GITR^+^ cells in Foxp3^−^CD4^+^ cells was determined by flow‐cytometric analysis (four animals from one experiment). (B) Violet Fluorescence‐labeled GITR^−^CD25^−^CD4^+^ cells (5 × 10^4^ cells) were cultured with unlabeled‐GITR^−^CD25^−^CD4^+^ cells, GITR^+^CD25^+^CD4^+^ cells, or GITR^+^CD25^−^CD4^+^ cells (2.5 × 10^4^ cells) in the presence of IA/IE^+^ cells (5 × 10^4^ cells) and anti‐CD3 mAb (1.0 μg/mL) for 72 h. Proliferation index was analyzed by FlowJo software (five animals from one experiment). (C) GITR^−^CD25^−^CD4^+^, GITR^+^CD25^+^CD4^+^ cells, and GITR^+^CD25^−^CD4^+^ were stimulated with anti‐CD3/anti‐CD28 coated dynabeads and IL‐2 (50 U/mL) for 72 h. The percentage of Foxp3^+^ cells and IL‐10^+^ cells and mean fluorescence intensity of CTLA‐4 expression were determined by flow‐cytometric analysis (three determinations). The results are shown as mean + SD. The significance of differences was examined by Duncan's test (*denotes *P *< 0.05).

Next, we examined the changes in non‐Treg cells by flow cytometry. Our results show that the proportion of GITR^+^ cells in Foxp3^−^CD4^+^ cells was significantly increased only in the FTY720 plus GPI_325‐339_ group (Fig. 5A). A previous report showed that the percentage of CTLA‐4^+^ cells in Foxp3^−^CD4^+^ cells was significantly increased by combination treatment 14. In addition, the proportion of CD39^+^ cells in Foxp3^−^CD4^+^ cells was modestly increased only in the FTY720 plus GPI_325‐339_ group (data not shown). The GITR^+^Foxp3^−^CD4^+^ cell population, that is, the GITR^+^ non‐Treg cell population, shared the CTLA‐4^+^ non‐Treg cell population; however, the GITR^+^ non‐Treg cell population did not share the CD39^+^ non‐Treg population.

To examine the suppressive activity of GITR^+^non‐Treg cells *in vitro*, a suppression assay was performed as described in the Materials and Methods section. The results of this assay show that the proliferation of GITR^−^CD25^−^CD4^+^ cells was suppressed in the presence of GITR^+^CD25^−^CD4^+^ (Fig. 5B). In addition, IL‐10 production was significantly higher in GITR^+^CD25^−^CD4^+^ cells than in GITR^+^CD25^+^CD4^+^ cells following stimulation with anti‐CD3/anti‐CD28 coated dynabeads and IL‐2 (Fig. 5C). Most GITR^+^CD25^−^CD4^+^ IL‐10 producing cells were Foxp3 negative. In addition, high expression of CTLA‐4 was observed in GITR^+^CD25^−^CD4^+^ and GITR^+^CD25^+^CD4^+^ cells following stimulation (Fig. 5C). Thus, GITR^+^CD25^−^CD4^+^ cells, which constitute the anergic population, might prevent relapse following resensitization *via* high level production of IL‐10; the GITR^+^ non‐Treg cells that were induced by the combination treatment might play a key role in the establishment of tolerance.

## Discussion

In rheumatoid arthritis, induction of antigen‐specific tolerance is more desirable than non‐antigen specific tolerance, since the former may lead to fewer adverse effects (such as opportunistic infections); however, the causative/pathogenic antigen is not always clear and relevant antigens show high variability between human patients. Immune tolerance induced by the administration of an antigen is highly specific and is an appealing method for preventing autoimmune diseases. Intravenous or intraperitoneal administration of antigens has been shown to successfully prevent autoimmune diseases in animal models, such as experimental autoimmune encephalomyelitis 22, 23; in these studies tolerance was induced as a result of anergy or clonal deletion of antigen‐specific T cells 22, 23. Previously, we reported that administration of the pathogenic antigen, GPI_325‐339_, in combination with FTY720 efficiently suppressed the progression of GPI_325‐339_‐induced arthritis symptoms by induction of: (1) T cell apoptosis; (2) inhibitory molecule (CTLA‐4 and programmed death‐1) expressing non‐Treg cells; and (3) the expansion of Treg cells in inguinal LNs 14. In this study, we demonstrated that the combination treatment could maintain remission, during which the immunological memory for regulation of pathogenic T cells might be efficiently induced, suggesting the involvement of unknown environmental and anergic mechanism(s) in tolerance maintenance.

GITR is overexpressed on CD25^+^CD4^+^ Treg cells and plays a key role in the maintenance of immunological self‐tolerance 17. Uraushihara et al. 24 reported that GITR^+^CD25^−^CD4^+^ cells: (1) exert suppressive activity in inflammatory bowel disease; (2) express elevated levels of CTLA‐4 as well as GITR^+^CD25^+^CD4^+^ cells; (3) suppress the proliferation of CD4^+^ cells *in vitro*; and (4) produce higher levels of IL‐10 and TGF‐β. Ono et al. 25 also reported that both CD25^+^CD4^+^ T cells and GITR^high^CD25^−^CD4^+^ cells possess autoimmune‐suppressive activity. These findings suggest that GITR plays a key role in the suppression of T‐cell responses. In contrast, Shimizu et al. 17 reported that naïve conventional T cells lead to GITR upregulation following activation and that CD25^−^CD4^+^ T cell‐derived GITR^high^ cells are not suppressive *in vitro*. In addition, GITR has been established as a marker for activated effector T cells rather than for Treg 26. These findings indicate that GITR‐expressing cells do not necessarily constitute suppressor cells. In this study, we demonstrated that GITR^+^CD25^−^CD4^+^ cells, which were induced by combination treatment with FTY720 plus pathogenic antigen *in vivo*, had a suppressive effect on T cell proliferation and exhibited high production potential for IL‐10 following stimulation with CD3/CD28. This indicates that GITR^+^CD25^−^CD4^+^ cells contribute not only to the induction of remission but also to its maintenance. GITR^+^CD25^−^CD4^+^ cells were not induced by administration of FTY720 alone or pathogenic antigen alone. These findings suggest that efficient *in vivo* induction of GITR^+^CD25^−^CD4^+^ cells, which possess suppressive activity, requires both robust stimulation and high cell density conditions in LNs.

Type 1 regulatory T (Tr1) cells are generally characterized by the production of high levels of IL‐10, moderate levels of TGF‐β, IFN‐γ and IL‐5, low levels of IL‐2, and no IL‐4 27, 28. Additionally, it has been reported that the surface markers, CD49b and lymphocyte activation gene 3, are stably and selectively coexpressed on human and mouse Tr1 cells 29. Asnagli et al. 30 reported that collagen type II‐specific Tr1 clones, which were expanded *in vitro* from collagen type II‐specific TCR transgenic mice, were characterized by a specific cytokine profile (IL‐10^high^IL‐4^neg^IFN‐γ^int^) and expression of GITR, granzyme B, CTLA‐4, and CD39. Moreover, adoptive transfer of Tr1 cells had a therapeutic effect on collagen‐induced arthritis 30. The function of GITR^+^CD25^−^CD4^+^ cells, which are induced by FTY720 plus pathogenic antigen, might be similar to that of the Tr1 cells. The key findings of this study are as follows: (1) combination treatment could efficiently induce Tr1‐like cells *in vivo*. Further studies will be required to elucidate the cytokine profile of these cells; (2) the percentage of activation marker expressing‐nTreg cells was increased in inguinal LNs of mice treated with FTY720. Treg cells are known to suppress the effector T cell function by: (1) production of IL‐10, IL‐35, or TGF‐β; (2) suppression of IL‐2; (3) the release of granzyme B; and (4) production of extracellular adenosine 31. Treg cells failed to suppress the proliferation of CD25^−^CD4^+^ cells across the membrane, in contrast with the marked suppression observed when the two populations were on the same side of the membrane 32. These results indicate that Treg cell‐mediated suppression is contact dependent. As we have already stated, FTY720 sequesters lymphocytes and creates a highly cellular environment in secondary lymphoid tissues. Thus, FTY720 might provide the conditions required for maximal Treg cell activity *in vivo* and promote the induction of immune tolerance.

In conclusion, the results of the present study suggest that the combination treatment with FTY720 and a pathogenic antigen efficiently induces complete remission of GPI_325‐339_‐induced arthritis by induction of a cell population with high suppressive capacity and creating the required conditions for Treg cell activity *in vivo*. Further studies will be required to establish in detail the mechanism(s) involved in the induction and maintenance of anergy. The combination of FTY720 plus pathogenic antigen might constitute a breakthrough treatment for the induction of complete remission not only in rheumatoid arthritis but also in other autoimmune diseases.

## Materials and Methods

### Animals and ethics statement

DBA/1 mice were purchased from Japan SLC Inc. (Shizuoka, Japan) and bred under specific pathogen‐free conditions. The mice were given γ‐ray‐irradiated food (CRF‐1; Oriental Yeast Co., Ltd., Tokyo, Japan) and distilled water ad libitum. This study was performed according to a protocol approved by the Institutional Animal Care Committee of Setsunan University (approval number: K15‐15). Throughout the experimental procedures, every effort was made to minimize the number of animals used and their suffering.

### Drug and peptides

2‐Amino‐2‐[2‐(4‐octylphenyl)ethyl]propane‐1,3‐diol hydrochloride (fingolimod; FTY720) was kindly provided by Yoshitomi Pharmaceutical Industries, Ltd (current company name: Mitsubishi Tanabe Pharma Corporation, Osaka, Japan). Peptide 325(IWYINCFGCETHAML)339 of human glucose‐6‐phosphate isomerase (hGPI_325‐339_) and peptide 325(IWYINCYGCETHALL)339 of mouse GPI (mGPI_325‐339_) were purchased from Eurofins Genomics K. K., (Tokyo, Japan).

### Antibodies

Horse‐radish peroxidase‐conjugated anti‐mouse IgG (H + L chain) polyclonal antibody was purchased from Medical and Biological Laboratories Co., Ltd. (Nagoya, Japan). Violet 500‐conjugated anti‐mouse CD4 mAb (clone: RM4‐5, 1:200), fluorescein isothiocyanate‐conjugated anti‐mouse GITR mAb (clone: DTA‐1, 1:100), phycoerythrin (PE)‐conjugated anti‐mouse CTLA‐4 mAb (clone: UC10‐4F10‐11, 1:100), and purified anti‐mouse CD3ϵ mAb (clone: 145‐2C11) were purchased from BD Biosciences (San Jose, CA, USA). Brilliant Violet 421‐conjugated anti‐mouse Foxp3 mAb (clone: FJK‐16s, 1:100), allophycocyanin‐conjugated anti‐mouse CD25 mAb (clone: PC61.5, 1:50), and PE‐conjugated anti‐mouse major histocompatibility complex Class II (IA/IE) mAb (clone: M5/114.15.2, 1:200) were purchased from eBioscience (San Diego, CA, USA). Allophycocyanin‐conjugated anti‐mouse CD39 mAb (clone: Duha59, 1:200), PE‐conjugated anti‐mouse Helios mAb (clone: 22F6, 1:100), and Alexa647‐conjugated anti‐mouse IL‐10 mAb (clone: JES5‐16E3, 1:100) were purchased from BioLegend, Inc. (San Diego, CA, USA).

### Induction of GPI_325‐339_‐induced arthritis

DBA/1 mice (7‐ to 8‐week‐old males) were immunized by intracutaneous injection of hGPI_325‐339_ (10 μg) with Freund's complete adjuvant containing *Mycobacterium tuberculosis* H37Ra (BD Biosciences) at the base of the tail on day 0. Pertussis toxin (200 ng; EMD Chemicals, Inc., Gibbstown, NJ, USA) was injected intraperitoneally on days 0 and 2 post immunization. In some experiments, mice were resensitized by intracutaneous injection of hGPI_325–339_ (10 μg) with Freund's incomplete adjuvant (BD Biosciences) in the back on day 32 after the first immunization. The clinical symptoms of arthritis in each limb were examined and graded according to a clinical arthritis score of 0–4, as previously described 33, 34. The clinical scores of the four limbs were totaled for each mouse, yielding a maximum score of 16.

### Treatment schedules

GPI_325‐339_‐induced arthritis mice were divided into four groups (Placebo, FTY720 alone, GPI_325‐339_ alone, and FTY720 plus GPI_325‐339_) and treated from the day of arthritis onset (days 9–10) for 5 days. In the placebo group, mice were given water (orally) together with phosphate‐buffered saline (intravenous administration; *i.v*.) every day. In the FTY20 alone group, mice were administered FTY720 in water (1.0 mg/kg, orally) together with phosphate‐buffered saline (*i.v*.) every day. In the GPI_325‐339_ alone group, mice were administered water (orally) together with hGPI_325‐339_ (10 μg, *i.v*.) every day. In the FTY720 plus GPI_325‐339_ group, mice were administered FTY720 in water (1.0 mg/kg, orally) together with hGPI_325‐339_ (10 μg, *i.v*.) every day (Fig. 1).

### Histochemical staining

At days 42–43 after the first immunization, front limb joints were removed and tissues were fixed with 10% buffered formalin solution (Wako Pure Chemical Industries, Ltd., Osaka, Japan) for 2 days. The fixed limbs were defatted with 90% ethanol at 4°C for 2 days and decalcified with K‐CX (Falma Co., Tokyo, Japan) at 4°C for 9 days. Tissues were processed, embedded in paraffin, and cut into 5 μm sections. The sections were stained with hematoxylin‐eosin using Mayer's Hematoxylin Solution (Wako Pure Chemical Industries, Ltd.). Synovial hyperplasia and lymphocyte infiltration of joints were histologically graded as follows: 1, normal; 2, mild; 3, moderate; 4, severe (histological score) 34, 35. The histological score was evaluated blindly by two investigators and mean values were calculated.

### Measurement of anti‐GPI_325‐339_ total IgG antibody titer

At days 42–43 after the first immunization, peripheral blood samples were collected for measurement of GPI_325‐339_ antibody titer by enzyme‐linked immunosorbent assay. 96‐well polystyrene microplates were coated with human or mouse GPI_325‐339_ (1.0 µg/mL) in 0.1 M sodium phosphate buffer (pH 7.5) containing 0.1% NaN_3_ (0.1 mL/well) at 4°C overnight. Following incubation, the microplates were washed four times with 10 mM sodium phosphate buffer (pH 7.0) containing 0.1 M NaCl (buffer P, 0.25 mL/well). Then, 10 mM sodium phosphate buffer (pH 7.0) containing 0.1 M NaCl, 0.1% bovine serum albumin (BSA, Nacalai Tesque Inc., Kyoto, Japan), and 0.1% NaN_3_ (buffer A) was added to the wells (0.25 mL/well) and incubated at 4°C for 4 h. Following incubation, buffer A was removed, serum samples previously diluted 10,000‐fold with buffer A were added to the wells (0.15 mL/well), and then incubated at 37°C for 3 h and 4°C overnight. The microplates were then washed as detailed above. Goat (anti‐mouse IgG H + L) Fab'‐horse‐radish peroxidase‐conjugate (Medical and Biological Laboratories Co., Ltd.), previously diluted 7500‐fold with buffer P, containing 0.1% BSA was added to the wells (0.15 mL/well) and incubated at 37°C for 3 h. Finally, the microplates were washed as described above and incubated with 50 mM sodium acetate buffer (pH 5.0) containing 7.5 mM o‐phenylenediamine (Nacalai Tesque Inc.), 0.025% BSA, and 0.025% H_2_O_2_ (0.1 mL/well) at 37°C for 10–20 min. The enzymatic reaction was terminated by the addition of 1.2 M H_2_SO_4_ containing 0.24% Na_2_SO_3_ (0.05 mL/well). Absorbance at 490 nm was measured with a plate reader (Model 680, Bio‐Rad Laboratories, Inc., Hercules, CA, USA).

### Flow cytometry analysis

Cells were obtained from inguinal LNs upon completion of treatment (day 14 after the first immunization) and were hemolyzed with a solution of Tris (hydroxymethyl) aminomethane‐buffered ammonium chloride buffer (0.16 M ammonium chloride: 0.17 M Tris = 9:1, pH 7.2). Cells were stained with fluorescence‐labeled anti‐mouse CD4, GITR, and CD39 mAbs at 4°C for 30 min. The cells were fixed and permeabilized using the Foxp3 Fixation/Permeabilization Buffer (eBioscience) and then stained with fluorescence‐labeled anti‐mouse Foxp3 and Helios mAbs at 4°C for 30 min. Flow‐cytometric analysis was performed with BD FACS Aria II (BD Biosciences).

### Suppression assay

Upon completion of treatment (day 14 after the first immunization), cells were obtained from inguinal LNs of FTY720 plus GPI_325‐339_ combination‐treated mice and hemolyzed as described above. Cells were stained with fluorescence‐labeled anti‐mouse CD4, IA/IE, GITR, and CD25 mAbs at 4°C for 30 min. IA/IE^+^, GITR^−^CD25^−^CD4^+^, GITR^+^CD25^+^CD4^+^, and GITR^+^CD25^−^CD4^+^ cells were sorted with a BD FACS Aria II. A portion of the GITR^−^CD25^−^CD4^+^ cells were stained with the CellTrace™ Violet Cell Proliferation Kit for flow cytometry (Molecular Probes, Eugene, OR, USA). Violet fluorescence labeled‐responder cells (GITR^−^CD25^−^CD4^+^ cells; 5 × 10^4^ cells) were added into a 96‐well U‐bottom plate and cultured with unlabeled‐GITR^−^CD25^−^CD4^+^ cells, GITR^+^CD25^−^CD4^+^ cells, or GITR^+^CD25^+^CD4^+^ cells (2.5 × 10^4^ cells) in the presence of IA/IE^+^ cells (5 × 10^4^ cells) and anti‐CD3 mAb (1 μg/mL) at 37°C under 5% CO_2_. After 72 h, violet fluorescence intensity was analyzed with a BD FACS Aria II (BD Biosciences). The proliferation index was analyzed using FlowJo software (Tree Star, Inc., Ashland, OR, USA).

### Stimulation and intracellular staining

Upon completion of treatment (day 14 after the first immunization), GITR^−^CD25^−^CD4^+^, GITR^+^CD25^+^CD4^+^, and GITR^+^CD25^−^CD4^+^ cells from inguinal LNs of the FTY720 plus GPI_325‐339_ combination‐treated mice were purified using a BD FACS Aria II as described above. The cells (5 × 10^4^ cells) were added into a 96‐well flat‐plate and then activated with Dynabeads® Mouse T‐Activator CD3/CD28 for T‐Cell Expansion and Activation (Thermo Fisher Scientific, Inc., Waltham, MA, USA) and 50 U/mL recombinant mouse IL‐2 (Miltenyi Biotec, Bergisch Gladbach, Germany) at 37°C under 5% CO_2_. After 72 h, the cells were restimulated for 5 hours at 37°C under 5% CO_2_ with phorbol 12‐myristate 13‐acetate (15 ng/mL) and ionomycin (750 ng/mL) in the presence of GolgiStop (BD Biosciences). The cells were fixed and permeabilized using the BD Pharmingen™ Transcription Factor Buffer Set (BD Biosciences) and then stained with fluorescence‐labeled anti‐mouse Foxp3, IL‐10, and CTLA‐4 mAbs at 4°C for 30 min. Flow‐cytometric analysis was performed with BD FACS Aria II (BD Biosciences).

### Statistical analysis

Statistical analysis was carried out using IBM SPSS Statistics Version 22 software. Differences between the groups were compared using by one‐way analysis of variance followed by Duncan's test. Pearson's correlation coefficient test was used to determine the correlations; *P *< 0.05 was considered statistically significant.

## Conflicts of Interest

None declared.
